# Calcium Phosphate Nanoparticle-Based Vaccines as a Platform for Improvement of HIV-1 Env Antibody Responses by Intrastructural Help

**DOI:** 10.3390/nano9101389

**Published:** 2019-09-27

**Authors:** Dominik Damm, Leonardo Rojas-Sánchez, Hannah Theobald, Viktoriya Sokolova, Richard T. Wyatt, Klaus Überla, Matthias Epple, Vladimir Temchura

**Affiliations:** 1 Institute of Clinical and Molecular Virology, Friedrich-Alexander University Erlangen-Nürnberg, 91054 Erlangen, Germany; 2 Inorganic Chemistry and Center for Nanointegration Duisburg-Essen (CeNIDE), University of Duisburg-Essen, 45141 Essen, Germany; 3 Department of Immunology and Microbial Science, The Scripps Research Institute, La Jolla, CA 92037, USA

**Keywords:** nano-vaccines, HIV-1 Env trimers, B-cell targeting, intrastructural help

## Abstract

Incorporation of immunodominant T-helper epitopes of licensed vaccines into virus-like particles (VLP) allows to harness T-helper cells induced by the licensed vaccines to provide intrastructural help (ISH) for B-cell responses against the surface proteins of the VLPs. To explore whether ISH could also improve antibody responses to calcium phosphate (CaP) nanoparticle vaccines we loaded the nanoparticle core with a universal T-helper epitope of Tetanus toxoid (p30) and functionalized the surface of CaP nanoparticles with stabilized trimers of the HIV-1 envelope (Env) resulting in Env-CaP-p30 nanoparticles. In contrast to soluble Env trimers, Env containing CaP nanoparticles induced activation of naïve Env-specific B-cells in vitro. Mice previously vaccinated against Tetanus raised stronger humoral immune responses against Env after immunization with Env-CaP-p30 than mice not vaccinated against Tetanus. The enhancing effect of ISH on anti-Env antibody levels was not attended with increased Env-specific IFN-γ CD4 T-cell responses that otherwise may potentially influence the susceptibility to HIV-1 infection. Thus, CaP nanoparticles functionalized with stabilized HIV-1 Env trimers and heterologous T-helper epitopes are able to recruit heterologous T-helper cells induced by a licensed vaccine and improve anti-Env antibody responses by intrastructural help.

## 1. Introduction

The unique characteristic of HIV-1 to infect activated CD4 T-cells requires alternative vaccination strategies that differ from the classical approaches used [1]. Polyfunctional serum antibodies against envelope glycoprotein (Env) of HIV-1 correlate with spontaneous HIV-1 control by elite controllers [2] and appear to be crucial for vaccine-mediated protection against HIV-1 [3,4,5]. However, limited breadth, poor persistence of antibody responses to Env, and potential enhancement of susceptibility to HIV infection by vaccine-induced HIV-specific T-helper cell responses compromise HIV-1 vaccines previously designed for clinical trials [6,7]. Recently we demonstrated how T-helper cells induced by a licensed vaccine against Tetanus toxoid (TT) can be harnessed to provide help for Env-specific B-cells in mice immunized with HIV-1 virus-like particles (VLP) containing T-helper epitopes of TT [8]. This “intrastructural help” [9] (ISH) can be explained by uptake of entire VLPs by Env-specific B-cells and subsequent presentation of epitopes from all proteins of the VLPs on their major histocompatibility complex class II (MHC-II) molecules to harness corresponding T-cell help [8,10].

Biodegradable calcium phosphate (CaP) nanoparticles have several advantages compared to biological and polymer-based nanoparticles and have been used for experimental vaccination during the past decade [11]. Previously we designed CaP nanoparticles covered with a model antigen in order to achieve efficient targeting and activation of cognate B-cells in vitro [12] as well as induction of humoral immune responses in vivo [13]. In animal models, where CD4 T-cells were genetically non-reactive to the surface antigen of the CaP nanoparticles, incorporation of the p30 peptide, a promiscuous (universal) T-helper epitope from TT [14], overcame the lack of functionally active CD4 T-cell epitopes [13]. These data indicate that CaP nanoparticle-based vaccines might be potentially used for improvement of HIV-Env antibody responses by the ISH approach.

A gp160 precursor of HIV-1 Env is proteolytically cleaved into non-covalently linked gp120 and gp41 subunits, which assemble into a trimer of heterodimers [15]. Historically, the initial candidate vaccines for immunization with Env-based antigens used monomeric gp120 [16]. Recent advances in design and manufacturing of soluble HIV-1 Env trimers stabilized in the closed pre-fusion state provide an antigenic form of Env that induces Env antibodies recognizing the quaternary conformation associated with neutralizing activity against autologous HIV-1 [15,16].

In this study, we therefore designed CaP nanoparticles with stabilized HIV-1 Env trimers coupled to the surface and universal T-helper peptides of TT in the core. These nanoparticles were used to demonstrate effects of ISH in mice previously immunized with a licensed vaccine against Tetanus toxoid.

## 2. Materials and Methods

### 2.1. Mice, Ethical Statement

Six- to eight-week-old female wild-type (wt) C57bl/6NRj (Bl6) mice (Janvier, Le Genest-Saint-Isle, France), as well as mice with transgenic B-cell receptors (BCR) specific for HIV-1 Env (PGT-121 mice [17], in-house breeding, kindly provided by Dr. M. Nussenzweig, The Rockefeller University, New York, NY, USA) were used in this study. Mice were accommodated in the animal facility of the Faculty of Medicine, FAU (Erlangen, Germany), in accordance with the national law and were handled according to instructions of the Federation of European Laboratory Animal Science Associations. All animal experiments were approved by an external ethics committee of the North Rhine-Westphalian Ministry for Nature, Environment and Consumer Protection (license AZ 84-02.04.2014. A191) and confirmed by the Government of Lower Franconia (license 55.2-2532-2-96).

### 2.2. Plasmids and VLP Production

The plasmid encoding for BG505 NFL2P gp140 [18] was used for expression and purification of stabilized Env trimers (see 2.3). We introduced recombinant transmembrane (TM) and cytoplasmic (CD) domains of the surface protein G from the vesicular stomatitis virus (VSV-G; GenBank Accession Number: GU177825.1) downstream of the sequence encoding for the soluble Env protein to elicit pseudo-typed, membrane-bound Env trimers (BG505 NFL2P gp140-GTMCD). We further introduced the nucleotide sequence ttcaacaacttcaccgttagcttctggctgcgcgttccgaaagtttctgcttcccacctggaa, that encodes for the TT-derived peptide p30 (FNNFTVSFWLRVPKVSASHLE) into the Gag open reading frame of the plasmid Hgpsyn [19] (Hgpsyn-TTp30) that contains the codon-optimized genetic information for the HIV-1 structural precursor protein (Gag) and viral enzymes (Pol). The p30 sequence was inserted between the p17 matrix protein and the p24 capsid protein. VLPs lacking Env (Δ-VLP), VLPs bearing BG505 NFL2P gp140-GTMCD Env trimers on the surface (Env-VLP) and Env-VLP with the Gag-p30 fusion protein inside (Env-VLP-p30) were produced by co-transfection of 293T cells with equal amounts of the corresponding plasmids and purified as described previously [20].

### 2.3. Production and Analyses of HIV-1 Env Trimers

#### 2.3.1. Production of HIV-1 Env Trimers

The HIV-1 subtype A envelope protein BG505 NFL2P gp140 (Env) was used as the primary antigen. In brief, this antigen consists of three heterodimeric gp120-gp41_ecto_ subunits and was stabilized for the soluble, trimeric conformation by truncation of the transmembrane and cytoplasmic domains, introduction of a flexible linker (2 x G4S) between the globular gp120 subunit and the gp41 ectodomain as well as a point mutation at amino acid position 559 (I559P) [18]. Env trimers were produced by transfection of 293F cells at a density of 1.0 × 10^6^ cells per mL with the plasmid encoding for BG505 NFL2P gp140 (1 µg/mL DNA). Linear polyethylenimine (PEI, Polysciences Inc., Warrington, PA, USA) was used as transfection reagent in a threefold excess compared to the mass of DNA. Cell culture medium was changed 6 h after the transfection. The supernatant was harvested after three days, sterile-filtered and run over a lectin affinity column (Agarose-bound *Galanthus nivalis* lectin, Vector Laboratories Inc., Burlingame, CA, USA). Env trimers were eluted using 1M Methyl-α-D-mannopyranoside and concentrated with a 10 kDa Amicon cutoff filter (Sigma-Aldrich, St. Louis, MO, USA). Between the centrifugation steps, buffer changes were performed by refilling with DPBS without bivalent cations (Thermo Fisher Scientific, Waltham, MA, USA). The final Env concentration was determined by photometric measurement with a nanodrop device (Thermo Fisher Scientific, Waltham, MA, USA). As a control, we introduced a stop codon downstream of the flexible linker and deleted the nucleotide sequence for the gp41 ectodomain in order to produce gp120 monomers. These proteins were purified in the same way as described above for the trimers. All purified proteins were analyzed by NativePAGE and western blot (WB). For the UV-Vis spectroscopy (see 2.4.4) Env trimers were labelled with AlexaFluor®-488 fluorescent dye using a protein labeling kit (Thermo Fisher Scientific, Waltham, MA, USA) following the manufacturer’s instructions.

#### 2.3.2. NativePAGE Analysis of HIV-1 Env Trimers

The native conformation of Env trimers and monomers was addressed using the NativePAGE system (Thermo Fisher Scientific, Waltham, MA, USA) following the manufacturer’s guidelines. In brief, 1 µg of each protein was mixed with G-250 additive and loaded onto a 4–16% Native Page Bis-Tris gel. The proteins were separated according to their native size by gel electrophoresis. Afterwards, excessive Coomassie stain was removed from the native gel by overnight fixation in 10% acetic acid and 30% ethanol in ultra-pure H_2_O. Faint protein bands on the gel were then developed by staining with silver nitrate using a Silver Stain Kit (Pierce Biotechnology Inc., Rockford, IL, USA). 

#### 2.3.3. Western Blot Analysis of HIV-1 Env Trimers

For the antigen-specific immunoblotting of purified Env, 1 µg of each soluble protein sample or 300 ng Env on VLPs were mixed with house-made, reducing SDS sample buffer, boiled and then loaded onto a 12% SDS gel. After gel electrophoresis, proteins were transferred onto a nitrocellulose membrane which was subsequently blocked with 5% skimmed milk in DPBS supplemented with 0.1% Tween20 (PBS-T). The blocked membranes were incubated with polyclonal goat anti-gp120 (Acris Antibodies GmbH, Herford, Germany) and horseradish peroxidase-coupled secondary anti-goat IgG antibody (Dianova, Hamburg, Germany) with multiple washing steps in between. The membranes were finally developed with house-made ECL solution and protein bands were imaged using an Advanced Fluorescence Imager (Intas, Göttingen, Germany).

### 2.4. Production of Calcium Phosphate Nanoparticles

#### 2.4.1. Instruments

Dynamic light scattering (DLS) and zeta potential were measured with a Zetasizer Nano ZS instrument (laser wavelength λ = 633 nm, Malvern Instruments, Malvern, UK) with the Smoluchowski approximation. Data obtained from Malvern software were used without further treatment; the particle size results refer to the z-average. Scanning electron microscopy (SEM) was performed with an ESEM Quanta 400 instrument (FEI Co., Hillsboro, OR, USA) and gold/palladium-sputtered samples. Calcium concentrations were measured by atomic absorption spectroscopy (AAS) with an M-Series AA spectrometer (Thermo Electron Corporation, Schwerte, Germany). UV-Vis absorption spectra were measured with a DS-11 FX+ spectrophotometer (“Nanodrop”, DeNovix, Wilmington, DE, USA) and a Cary 300 Bio spectrophotometer (Agilent Technologies, Santa Clara, CA, USA). Ultracentrifugation was done at 20 °C with a Sorvall WX Ultra Series centrifuge (Thermo Electron Corporation, Schwerte, Germany). Freeze-drying (lyophilization) was carried out with a Christ Alpha 2-4 LSC instrument (Martin Christ GmbH, Osterode am Harz, Germany). The endotoxin concentration was measured with an Endosafe Nexgen-PTS handheld spectrophotometer (Charles River, Boston, MA USA). Ultrapure water (Purelab, ELGA LabWater, Celle, Germany) was used for all preparations. All nanoparticles were prepared and analyzed at room temperature.

#### 2.4.2. Synthesis of Calcium Phosphate Nanoparticles

The CaP nanoparticle synthesis was performed according to our previously described method [21]. In brief, aqueous solutions of calcium lactate (18 mM, pH = 10, p.a., Sigma-Aldrich Corp., St. Louis, MO, USA), diammonium hydrogen phosphate (10.8 mM, pH = 10, p.a., VWR Life-Sciences) and branched polyethyleneimine (PEI, Mw = 25 kDa, Sigma-Aldrich Corp., St. Louis, MO, USA) were simultaneously pumped at a volume ratio of 5:5:7 mL during one minute into a stirred vessel with 20 mL of ultrapure water. The dispersion was stirred for 20 min. and the CaP/PEI nanoparticle dispersion was used immediately for the following steps. For the synthesis of adjuvant-containing nanoparticles, 1 mL CaP/PEI nanoparticle dispersion was mixed with aqueous solutions of either 60 µL p30 peptide (1 mg/mL) or 40 µL CpG (1 mg/mL) under stirring, followed by 30 min. stirring at room temperature (RT). The adjuvant loading was determined by measuring the residual concentration in the supernatant by UV microvolume spectroscopy (“nanodrop”). For further surface modifications a silica shell was added to the nanoparticles. To this end, 1 mL of either CaP/PEI or of adjuvant-containing nanoparticle dispersion was added to a mixture of 4 mL ethanol (Fisher Chemicals, Hampton, NH, USA), 5 µL tetraethylorthosilicate (TEOS, Sigma-Aldrich Corp., St. Louis, MO, USA) and 10 µL aqueous ammonia solution (7.8 wt.%) and stirred for 16 h. After this time, the nanoparticles were isolated by ultracentrifugation (66,000× *g*, 30 min, 20 °C) and redispersed with 1 mL ultrapure water followed by ultrasonication (UP50H, Hielscher, Teltow, Germany, sonotrode MS7m cycle 0.8, amplitude 70%, 4 s). Silica-terminated calcium phosphate nanoparticles (CaP/PEI/SiO_2_) were obtained. To prepare the thiol-terminated calcium phosphate nanoparticles a surface modification with (3-mercaptopropyl)trimethoxysilane (MPS, Sigma-Aldrich Corp., St. Louis, MO, USA) was performed. For this, 1 mL of CaP/PEI/SiO_2_ nanoparticles was added to a mixture of 4 mL ethanol and 50 µL (3-mercaptopropyl)trimethoxysilane and stirred for 8 h. The nanoparticles were isolated by ultracentrifugation (66,000× *g*, 30 min, 20 °C), redispersed with 1 mL H_2_O and ultrasonicated. After this step, thiol-terminated calcium phosphate nanoparticles (CaP/PEI/SiO_2_-SH) were obtained.

#### 2.4.3. Functionalization of CaP Nanoparticles with Env Trimers

The Env trimers (Mw = 140 kDa) were coupled to the nanoparticle surface via a sulfo-SMCC cross-linker (sulfosuccinimidyl-*trans*-4-(N-maleimidomethyl)cyclohexane-1-carboxylate, Merck, Darmstadt, Germany). One end of the linker reacts with the primary amines in Env and the other end with the thiol groups on the nanoparticle surface. For this, 300 µL aqueous sulfo-SMCC solution (1.78 mg/mL) were given to 600 µL Env in DPBS (1 mg/mL) and incubated for 2 h at 4 °C. After incubation, the protein was purified from the unreacted cross-linker with a 3 kDa Amicon ultracentrifuge filter (Merck, Darmstadt, Germany), following the manufacturer recommendations. To attach the Env trimers to the nanoparticle surface, 330 µL activated protein (1 mg/mL) were given to 4 mL CaP/PEI/SiO_2_-SH nanoparticle dispersion and incubated for 24 h at 4 °C. After this time, the particles were isolated by centrifugation at 21,000× *g* and 8 °C and washed once with 1 mL H_2_O. Finally, the nanoparticles were redispersed in 4 mL H_2_O and ultrasonicated (UP50H, Hielscher, sonotrode MS7m cycle 0.8, amplitude 70%, 4 s). The different syntheses were carried out with sterile-filtered solutions.

#### 2.4.4. CaP nanoparticle Characterization and Storage

For the final nanoparticle dispersion, an endotoxin quantification assay was performed with an Endosafe Nexgen-PTS device. No endotoxin was detected in all synthesized nanoparticles (<0.1 EU/mL). To calculate the number of nanoparticles per volume unit, the Ca2^+^ concentration was measured by AAS and then tentatively expressed as the most common calcium phosphate, i.e., hydroxyapatite, Ca_10_(PO4)_6_(OH)_2_. The nanoparticle concentration in 1 mL dispersion is calculated from the measured calcium phosphate concentration and the density of hydroxyapatite (3140 kg/m^3^) assuming a complete spherical morphology. Additionally, the number of Env units on the nanoparticle surface was determined by UV-Vis spectroscopy with an AlexaFluor®-488 labelled Env protein and UV microvolume spectroscopy (“Nanodrop”) (see [22] for typical calculation steps to obtain these data). For storage and transportation, the nanoparticle dispersion was lyophilized according to our previously reported protocol [23]. 20 mg D-(+)-trehalose dihydrate (Sigma-Aldrich Corp., St. Louis, MO, USA) were added to 1 mL of the nanoparticle dispersions as cryoprotectant followed by shock-freezing with liquid nitrogen and lyophilization for 72 h at 0.31 mBar and −10 °C. Immediately before the application, the nanoparticles were redispersed in 1 mL ultrapure water and gently sonicated with an ultrasonication bath.

### 2.5. B-cell Activation In Vitro 

B-cells from wt Bl6 mice or PGT-121 BCR-transgenic mice were isolated from the spleen by magnetic cell separation (Miltenyi Biotec, Bergisch Gladbach, Germany, #130-090-862,). 2.0 × 10^5^ cells were incubated with different concentrations of Env-coupled nanoparticles in U-bottom 96-well plates. As controls, we additionally incubated B cells with soluble Env trimers, with Env-VLP (0.2 µg of Env/mL) and with 2 µg/mL of LPS (Sigma-Aldrich, Corp., St. Louis, MO, USA). After 18 h incubation at 37 °C and 5% CO_2_, cells were stained with Fixable Viability Dye (Thermo Fisher Scientific, Waltham, MA, USA) and with antibodies against the B-cell surface antigen CD19 (Thermo Fisher Scientific, Waltham, MA, USA) and the early activation marker CD69 (Thermo Fisher Scientific, Waltham, MA, USA). B-cell activation in living B-cells was subsequently measured on a benchtop flow cytometer BD™ LSR II (BD Biosciences, Franklin Lakes, NJ, USA) and analyzed with the FlowJo software (BD Biosciences, Franklin Lakes, NJ, USA).

### 2.6. Analyses of In Vivo Induced Immune Responses

#### 2.6.1. Immunization, Collection of Blood and Organ Samples

All immunizations were performed intramuscularly in both hind legs in Bl6 mice that were at the age of 6–8 weeks by the time of the first injection. For the induction of intrastructural help, mice were immunized on day 0 and day 28 with Tetanus toxoid vaccine (Tetanol® pur, GSK Vaccines GmbH, Marburg, Germany) diluted 1:10 in sterile DPBS to induce CD4 T-cell responses against the TT peptide p30 (ISH group) or with DPBS alone (control group). All mice were then boosted on day 56, day 84 and day 112 with one of the following particle types: Env trimer-coupled CaP nanoparticles (Env-CaP); Env-CaP with encapsulated p30 peptide (Env-CaP-p30); or Env-VLP-p30 with the membrane-bound form of the Env trimer on the surface and the Gag-p30 fusion protein inside. To evaluate the influence of encapsulated CpG as an adjuvant, mice were boosted thrice with Env-CaP-CpG particles in the same immunization protocol as described above. The injection doses were normalized to 10 µg of Env delivered with CaP nanoparticles and 300 ng of Env delivered with VLPs per mouse. To investigate humoral immune responses against Env, blood samples were collected 2 weeks after each particle immunization. To investigate humoral immune responses against TT, blood samples were collected on day 49. All blood samples were taken under isoflurane anesthesia from the retrobulbar venous plexus with non-heparinized, single-use capillaries (minicaps®, Hirschmann, Eberstadt, Germany). Collected blood samples were centrifuged for 5 min at 5000 rpm. The upper serum fractions were isolated and stored at −20 °C. Mice were sacrificed on day 126 and spleens were isolated for further assessment of the cellular immune response against both Env and p30.

#### 2.6.2. Analyses of Humoral Immune Responses

Serum ELISAs to address the humoral immune response against Env were performed as described previously [8]. Briefly, white opaque MaxiSorp 96-well plates (Greiner Bio One, Frickenhausen, Germany) were coated with 100 ng Env trimer in coating buffer (0.1 M Na_2_CO_3_, 0.1 M NaHCO_3_ in H_2_O, pH 9.6) per well at 4 °C overnight. After 1 h blocking with 5% skimmed milk (diluted in DPBS containing 0.05% Tween-20), the wells were incubated with serum from immunized mice diluted 1:1000 in 2% skimmed milk for 1 h at RT. Plates were washed and then incubated for 1 h at RT with 1:4000 dilutions of HRP-coupled secondary antibodies specific for different murine IgG subtypes as well as total IgG (Southern Biotech, Birmingham, AL, USA). After thorough washing, serum binding was detected by addition of ECL solution and measurement of the relative light units per second (RLU/s) with an Orion microplate luminometer (Berthold Detection Systems GmbH, Pforzheim, Germany).

#### 2.6.3. Analyses of Cellular Immune Responses

For the analysis of the cytokine profiles of both Env- and p30-specific CD4 T-cells after immunizations, we performed in vitro intracellular cytokine staining (ICS) of CD4 T-cells for interferon gamma (IFN-γ), tumor necrosis factor alpha (TNF-α) and interleukin-2 (IL-2), as well as cytokine ELISA for IL-5. Isolated spleens were dissociated by using a gentleMACS™ Dissociator (Miltenyi Biotech, Bergisch Gladbach, Germany) following the manufacturer’s guidelines. The cell suspensions were run through 70 µm cell strainers. We removed erythrocytes by incubation with ACK lysis buffer (150 mM NH_4_Cl, 10 mM KHCO_3_, 0.1 mM EDTA in H_2_O, pH 7.2) for 8 min at RT; the lysis reaction was stopped by the addition of R10 medium (RPMI 1640, 10% FCS, 1% Penicillin-Streptomycin, 10 mM HEPES, 2 mM L-glutamine, 50 µM 2-mercaptoethanol). The isolated splenocytes were washed twice by centrifugation and resuspension in R10 and subsequently counted using a Countess Automated Cell Counter (Thermo Fisher Scientific, Waltham, MA, USA ). Isolated splenocytes were seeded in 96-well U-bottom plates (1.0 × 10^6^ cells per well). For antigen-driven cytokine production by p30-specific CD4 T-cells, splenocytes were re-stimulated with 5 µg/mL of p30 peptide. Since there is no identified immunodominant MHC-II restricted Env peptides for Bl6 mice, we established a re-stimulation protocol to induce Env-specific cellular responses. To this end, we purified PGT121 B-cells (see above) and incubated them for 3 h at 37 °C with Env-VLPs containing 0.2 µg/mL Env or with the same amount of Δ-VLP. Thereafter, the B-cells, that were supposed to take up the Env-VLP in a BCR-dependent manner and subsequently present an array of different Env peptides to the CD4 T-cells, were washed with R10 and added to splenocyte suspensions (1.5 × 10^5^ PGT121 B-cells per well).

##### Intracellular Cytokine Staining

Antigen re-stimulated and unstimulated (control) splenocytes were incubated in the presence of 2 µg/mL anti-mouse CD28 antibody and 3 µg/mL Brefeldin A (eBioscience, San Diego, CA, USA) for 6 h at 37 °C and 5% CO_2_. After incubation, cells were washed with FACS buffer (1% FCS, 1 mM EDTA in DPBS without bivalent cations) and stained with anti-mouse CD4 antibody (eBioscience, San Diego, CA, USA) and Fixable Viability Dye (Thermo Fisher Scientific, Waltham, MA, USA). Then, the cells were fixated with 2% paraformaldehyde in DPBS and subsequently permeabilized using 0.5% Saponin in FACS buffer and intracellularly stained with anti-mouse IL-2, IFN-γ and TNF-α (eBioscience, San Diego, CA, USA). The cells were washed twice with permeabilization buffer and twice with FACS buffer. The cytokine accumulation in the CD4 T-cells was then analyzed by flow cytometry. The background values of unstimulated cultures (Δ-VLP for Env) were subtracted for each individual mouse.

##### Cytokine ELISA

Antigen re-stimulated and control splenocytes were incubated in the presence of 2 µg/mL anti-mouse CD28 antibody for 60 h at 37 °C and 5% CO_2_. The culture media were harvested and the IL-5 cytokine secretion was analyzed using a Mouse IL-5 ELISA kit (Invitrogen, Carlsbad, CA, USA) following the manufacturer’s instructions. The background values of unstimulated cultures were subtracted for each individual mouse.

### 2.7. Statistical Analysis

Statistical analyses were performed as indicated in the figure legends with the GraphPad Prism 7 software (Graphpad Software Inc., San Diego, CA, USA).

## 3. Results and Discussion

### 3.1. Production and Characterization of Soluble HIV-1 Env Trimers and Env-VLP-p30

For the coupling onto the surface of the CaP nanoparticles we needed faithful mimetics of the HIV-1 Env spike. During the last decade, there were great efforts to create soluble Env trimers that are stabilized in a closed pre-fusion conformation. This was achieved by truncation of the protein at amino acid position 664 within the gp41 subunit as well as by the introduction of various stabilizing mutations i.e., I559P [24].

**Figure 1:**
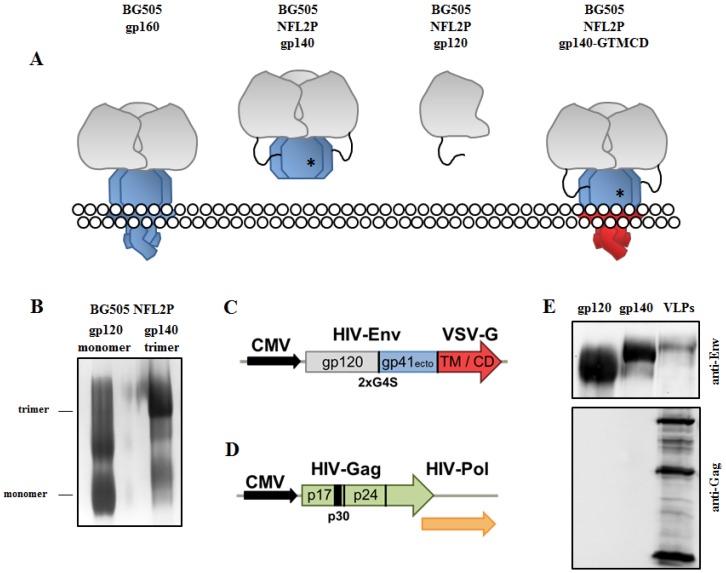
Production and characterization of soluble HIV-1 Env constructs and Env-p30-VLPs. (**A**) Overview of different membrane-embedded and soluble Env constructs. The native BG505 gp160 (left), that is composed of three gp120 (grey) – gp41 (blue) heterodimers, has been used previously to create a soluble stabilized recombinant trimer (BG505 NFL2P gp140; middle left) by truncation at amino acid 664 and introduction of both a point mutation (I559P) indicated with an asterisk (*) and a flexible linker (2xG4S). As a purification control, we produced a BG505 NFL2P gp120 monomer (middle right) by truncation of the protein downstream of the flexible linker. A recombinant VSV-G transmembrane and cytoplasmic domain (TM/CD; red) was introduced downstream of the gp41 ectodomain to create a membrane-bound form of the stabilized trimer for production of Env-VLP-p30 particles (BG505 NFL2P gp140-GTMCD; right). (**B**) Native PAGE of purified gp140 and gp120. Three gp140 molecules formed a globular trimer molecule represented by a major band at 700 kDa. The gp120 subunit was expressed as monomeric or dimeric proteins, but did not form trimers. (**C**,**D**) Schematic overview of the plasmids used for Env-VLP-p30 production. (**C**) BG505 NFL2P gp140-GTMCD construct. Colors are matched to their respective protein domains in Figure 1A. (**D**) Hgpsyn-TTp30 construct: the nucleotide sequence for the p30 peptide was introduced between the HIV-1 p17 matrix protein and the spacer protein 1 followed by the capsid protein p24. (**E**) Reducing SDS-PAGE and Western Blot of purified soluble Env proteins and Env-VLP-p30.

In this study, we used an Env trimer that is derived from the membrane-embedded native form (Figure 1A; left) of a subtype A HIV-1 isolate from an infected baby (BG505). In addition to the modifications mentioned above, a flexible linker (2xG4S) was more recently introduced between the gp120 subunit and the gp41 ectodomain to overcome the need for proper cleavage of the precursor protein in order to receive stabilized well-folded proteins [18].

We expressed these Env trimers (BG505 NFL2P gp140) (Figure 1A, middle left) as well as monomeric gp120 subunits (BG505 NFL2P gp120) (Figure 1A, middle right) in 293F cells and purified them by Lectin affinity chromatography. The purified and concentrated proteins were analyzed on a native gel followed by a silver staining. A prominent band at approximately 700 kDa represented the globular native form of an Env trimer that is formed by three non-covalently assembled gp120-gp41_ecto_ (gp140) heterodimers (Figure 1B). Two minor bands showed a low percentage of gp140 dimers and monomers. The purified gp120 resulted in two major bands on the native gel: a monomeric protein at 200 kDa and a gp120 dimer at 480 kDa that is formed by aberrant intermolecular di-sulfide bridges [25] as well as a large aggregate fraction (Figure 1B). As expected, the expression of gp140 (but not of gp120) results in the production of Env trimers, since the trimerization domain is located in the gp41 ectodomain and stabilized by the I559P mutation.

To compare intrastructural help for CaP nanoparticles side by side with that for HIV-1 VLPs, we generated VLPs with a membrane-bound form of the stabilized Env trimer on the surface and the TT-derived peptide p30 inside (Env-VLP-p30). In order to array stabilized BG505 NFL2P gp140 trimers on HIV-1 VLPs, the transmembrane and cytoplasmic domains of the vesicular stomatitis virus G-protein (VSV-G) were fused to the open reading frame encoding Env. Figure 1C represents the BG505 NFL2P gp140-GTMCD expression construct and the corresponding protein (Figure 1A; right) has an estimated size between 150 and 160 kDa. To produce HIV-1 VLPs that contain the p30 peptide of TT, we inserted the coding sequence for the peptide in frame between the open-reading frame for matrix and capsid of the HIV-1 structural Gag protein. Figure 1D represents the Hgpsyn-TTp30 construct. Env-VLPs-p30 particles were produced by co-transfection of 293T cells with both the BG505 NFL2P gp140-GTMCD and the Hgpsyn-TTp30 construct.

The purified Env proteins as well as the produced Env-VLP-p30 particles were analyzed by Western Blot under reducing conditions (Figure 1E). gp120 and gp140 were represented by prominent bands in the respective sizes. The pseudotyped BG505 NFL2P gp140-GTMCD protein was approximately 150 to 160 kDa in size and, therefore, runs slightly higher than the soluble trimer (Figure 1E; upper panel). In addition, three major Gag bands represent different states of the capsid maturation of the HIV-VLPs (Figure 1E; lower panel).

These characterized Env trimers were further used for the coupling onto the surface of the CaP nanoparticles.

### 3.2. Design, Production and Characterization of CaP Nanoparticles Functionalized with Soluble HIV-1 Env Trimers

**Figure 2:**
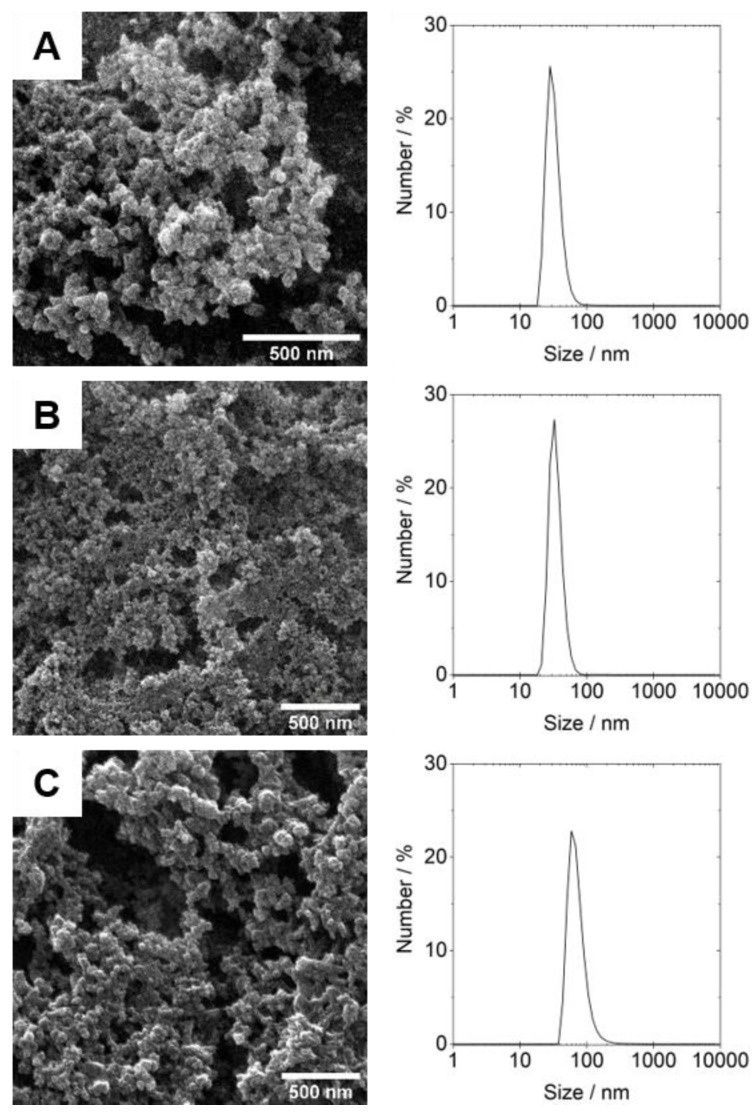
Scanning electron micrographs and dynamic light scattering (DLS) particle size distribution of the prepared nanoparticles: (**A**) Env-CaP, (**B**) Env-CaP-p30, (**C**) Env-CaP-CpG.

Stable CaP nanoparticles were synthesized and the surface modified with soluble stabilized HIV-1 Env trimer proteins (Figure 1). The obtained nanoparticles had an average solid core diameter between 38-57 nm (as determined by SEM) (Figure 2) with a positively charged surface near +27 mV, due to the stabilizing PEI polymer, and a hydrodynamic diameter between 300–400 nm. These parameters make the nanoparticles suitable for cellular uptake [26]. The complete characterization data are shown in Supplementary Table S1. For nanoparticles loaded with the T-helper peptide p30 or with CpG, the load was absorbed onto the nanoparticle core before forming the external silica shell around it. This coating provides protection to the internal loading but also permits a covalent surface modification with Env [21].

To make the protein reactive to the thiol-terminated calcium phosphate nanoparticles Env was activated with the crosslinker sulfo-SMCC by which the primary amines from the protein react with the N-hydroxysuccimide group. In the protein, 33 lysine residues are expected to have the side chain exposed to the solvent and can react with the NHS ester [27,28]. After the addition of the nanoparticles the maleimide group at the other end of the crosslinker reacts with the thiol groups on the nanoparticle surface to form a stable covalent bond between the nanoparticle and the protein.

To determine the coupling efficiency of the Env proteins to the nanoparticles, an AlexaFluor®-488 labelled Env protein was used for the surface modification. After attaching this labelled protein, the reaction yield was determined to 85% by UV-Vis spectroscopy. This factor was assumed to calculate the concentration of Env after attaching the unlabelled protein under equivalent reaction conditions. Additionally, a comparison for the Env concentration determined by nanodrop was performed. The measured concentrations are shown in Supplementary Table S1. The Env concentration determined by UV-Vis spectroscopy was used to calculate Env concentrations for all subsequent nanoparticle preparations.

### 3.3. Antigen-Specific Activation of Naïve B-Cells with Env-CaP Nanoparticles In Vitro

Previously we demonstrated in vitro that CaP nanoparticles functionalized with a monovalent model antigen activated naïve antigen-specific B-cells in a dose-dependent manner more efficiently than the dissolved antigen alone [12]. In contrast to monomeric proteins, each stabilized soluble Env trimer exposes up to 3 identical epitopes per molecule [29].

**Figure 3:**
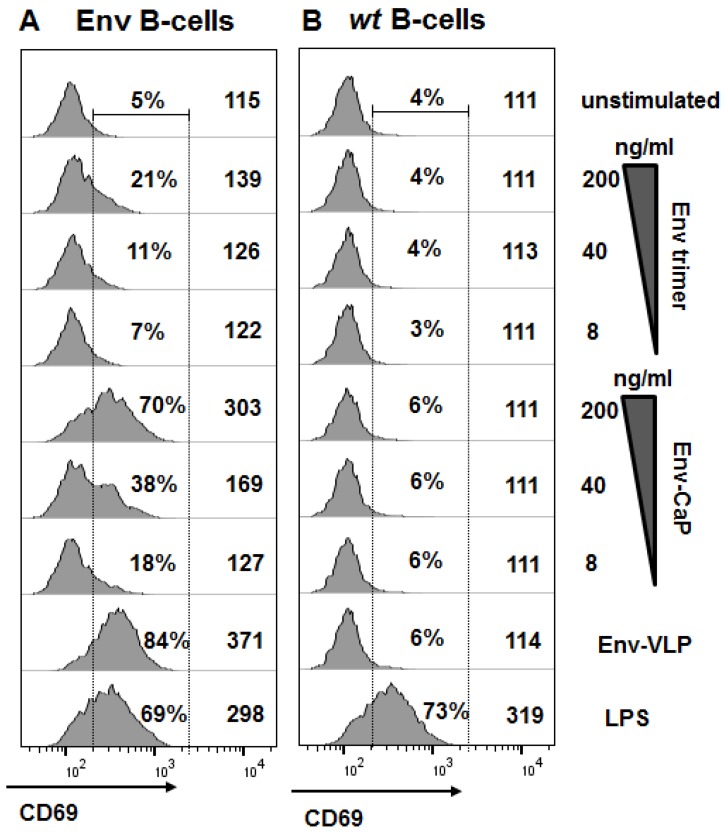
Activation of naïve B-cells in vitro. Naïve B-cells were isolated from PGT121 (**A**) or wt (**B**) mice and stimulated with Env trimers (8, 40 or 200 ng of Env/mL), Env-CaP nanoparticles (8, 40 or 200 ng of Env/mL), Env-VLPs (200 ng of Env/mL), or LPS (2 µg/mL). After 18h incubation, the cells were stained with a viability dye and with anti-CD19 and anti-CD69 antibodies. The histograms represent the expression of CD69 on the surface of viable CD19-positive B-cells. The numbers on the histograms indicate the percentage of the gated CD69-positive cells and medians of the total CD69 fluorescence intensity.

To investigate how the surface functionalization of CaP nanoparticle with stabilized HIV-1 Env trimers influences the activation of naïve Env-specific B cells, we incubated B-cells from transgenic mice that express the human PGT121 antibody [30] as B-cell receptors (Env B-cells) with Env-CaP and soluble Env trimers (Figure 3A). Env-VLPs served as a positive control for BCR-specific B-cell activation [31]. The stimulation with LPS showed the total polyclonal capability of the B-cells to be activated [32]. Within a concentration range of 8 ng to 200 ng of Env per 1 mL of culture medium, Env trimers arrayed on the CaP nanoparticle surface were able to activate Env-specific B cells more efficiently in a dose-dependent manner than soluble trimers in the same concentrations (Figure 3A).

Incubation of wt B-cells in the presence of Env-CaP nanoparticles, soluble Env trimers or Env-VLPs did not reveal Env-specific early B-cell activation (Figure 3B). The polyclonal activation of wt B-cells with LPS (comparable to those of Env-specific B-cells) indicated the functional activity of the wt B-cells (Figure 3) and, therefore, clearly demonstrates that all experimental Env preparations used were free of polyclonal activators. Our results are consistent with the data reported by Ingale et al., who showed that HIV-1 trimer-conjugated liposomes activated Env-specific B-cells better than soluble trimers [33].

Thus, the surface functionalization of CaP nanoparticles with soluble HIV-1 Env trimers improved the dose-dependent activation of naïve Env-specific B-cells in vitro in comparison to soluble HIV-1 Env trimers alone.

### 3.4. Improvement of HIV-1 Env Antibody Responses by Intrastructural Help

**Figure 4:**
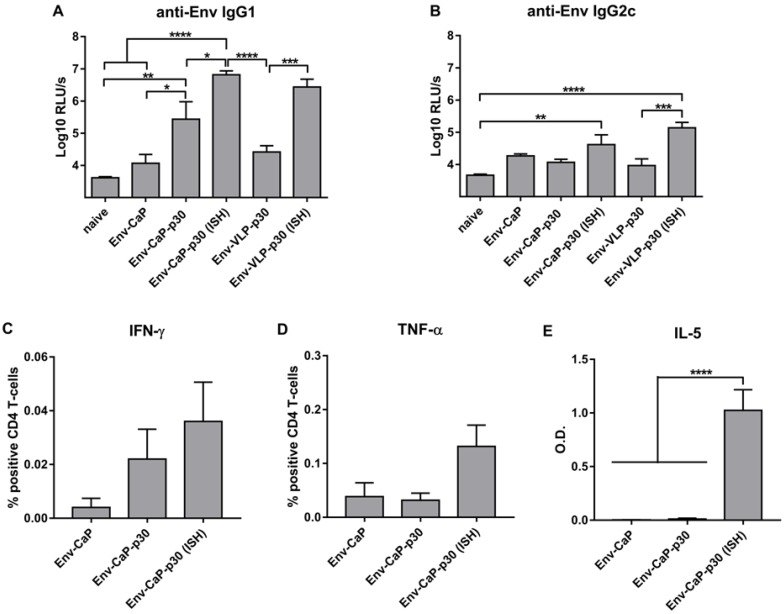
Improvement of Env-specific IgG subtype responses after Env-CaP-p30 immunizations by intrastructural help from Tetanus toxoid-specific T-helper cells. (**A**,**B**) IgG1 (**A**) and IgG2c (**B**) Env-specific antibody responses were measured in sera from wt mice primed twice with DPBS or Tetanol (ISH) and boosted 3 times with different CaP nanoparticles or Env-VLP-p30. (**C**,**D**) Percentages of CD4 T-cells producing IFN-γ (**C**) and TNF-α (**D**) from differently immunized wt animals after in vitro re-stimulation with p30 peptide were measured by intracellular cytokine staining. (**E**) p30-specific IL-5 cytokine secretion as determined by ELISA. The columns represent the mean values of six animals ± SEM. * *p* < 0.05; ** *p* < 0.001; *** *p* < 0.0005; **** *p* < 0.0001; one-way ANOVA with Tukey multiple comparison post-hoc test.

To analyze the induction of anti-Env antibody responses in vivo, we applied Env-CaP nanoparticles intramuscularly, as it was demonstrated to be the most appropriate way for the delivery of B- and T-cell antigens with CaP nanoparticles into draining lymphoid organs [13]. In contrast to the model antigen [13] in a highly reactive C3H mouse strain (as discussed in Reference [34]), Env-CaP nanoparticles at a dose of 10 µg of Env protein per immunization induced poor anti-Env antibody responses in Bl6 mice (Figure 4A, Env-CaP vs. naive).

Although Env-CaP nanoparticles are able to directly activate Env-specific B-cells in vitro (Figure 3), the primary anti-Env antibody response in vivo is also dependent on CD4 T-cell help [35]. The low magnitude of Env-specific IgG antibody responses induced with Env-CaP might be due to (i) a suboptimal MHC-II restricted T-cell help [14] elicited by HIV-1 BG505 Env protein in Bl6 mice [36], or (ii) suboptimal immunogenic capacities of Env-CaP (as discussed in [13]).

We have already demonstrated that the functionalization of CaP-HEL nanoparticles with the p30 peptide of TT (a universal T-helper epitope [14]) overcomes the lack of functionally active HEL-derived CD4 T-cell epitopes in Bl6 mice [13]. The functionalization of Env-CaP nanoparticles with the p30 peptide increased the magnitude of the anti-Env IgG1 antibody response (Figure 4A, Env-CaP vs. Env-CaP-p30), indicating an initially suboptimal MHC-II restricted T-cell help in mice immunized with Env-CaP. The anti-Env IgG2c antibody response, however, remained unaffected (Figure 4B, Env-CaP vs. Env-CaP-p30).

To further improve the Env-CaP-p30 induced anti-Env humoral immune responses, we recruited pre-existing T-helper cells generated by the licensed Tetanol pur vaccine to provide ISH for Env-specific B-cells. Mice previously vaccinated against Tetanus demonstrated significantly higher anti-Env antibody responses after Env-CaP-p30 nanoparticle immunization, than the control animals injected with DPBS instead of Tetanol pur (Figure 4A,B; Env-CaP-p30 vs. Env-CaP-p30 (ISH)). The in vitro reactivation of p30-specific CD4 T-cells from the spleens of differently immunized mice revealed an increase of potential heterologous T-cell help in the ISH group (Figure 4C–E). Intracellular cytokine staining for the Th1 cytokines [37] IFN-γ (Figure 4C) and TNF-α (Figure 4D) showed a trend of being more pronounced in the ISH group. The secretion of the Th2 cytokine [37] IL-5 after p30 reactivation was significantly higher in the ISH group (Figure 4E). CD4 T-cells that produce either Th1 or Th2 cytokines support the generation of IgG2a (IgG2c in Bl6 mice) and IgG1 antibody subclasses in mice, respectively [37,38]. The prominent Th2 cytokine profile of p30-specific CD4 T-cells is, therefore, consistent with the predominant induction of the anti-Env IgG1 antibody subclass after immunizations with Env-CaP-p30 nanoparticles (Figure 4A,B).

Previously, we demonstrated ISH in mice immunized with Tetanol pur prior to administration of HIV-1 derived VLPs containing different T helper epitopes of TT [8]. For direct comparison of the ISH effects on the anti-Env antibody induction between the CaP nanoparticle platform and the HIV-1 VLP system, we produced VLPs containing the p30 epitope of TT within Gag (Figure 1D) and presenting stabilized BG505 Env trimers in a membrane-anchored form on their surface (Figure 1A,C). The VLP antigen dosage was selected based on our previous studies [8,35] and was 0.3 µg of Env protein per immunization. For the Env-VLP-p30 particles we observed intrastructural help (Figure 4A,B; Env-VLP-p30 vs. Env-VLP-p30 (ISH)) in a magnitude comparable to the CaP nanoparticle platform (Figure 4A,B; Env-VLP-p30 (ISH) vs. Env-CaP-p30 (ISH)).

Altogether, the incorporation of T-helper cell epitopes of non-HIV proteins into CaP nanoparticles functionalized with HIV-1 Env trimers may allow the Env-specific B-cells to get T-cell help from non-HIV specific CD4 T-cells via the ISH mechanism.

### 3.5. Distinct Effects of ISH and CpG-Adjuvants on the Induction of Env-Specific CD4 T-Cell Responses

Along with engaging the heterologous universal p30 T-helper epitope, functionalization of the B-cell targeting CaP-nanoparticles with TLR-ligands significantly increased IgG antibody responses against a model antigen in mice [13]. The TLR9 ligand CpG demonstrated a number of advantages to other TLR-ligands tested in the study (as discussed in Reference [13]). In addition, the CpG-based adjuvant 1018 ISS [39] is already approved for clinical use.

**Figure 5:**
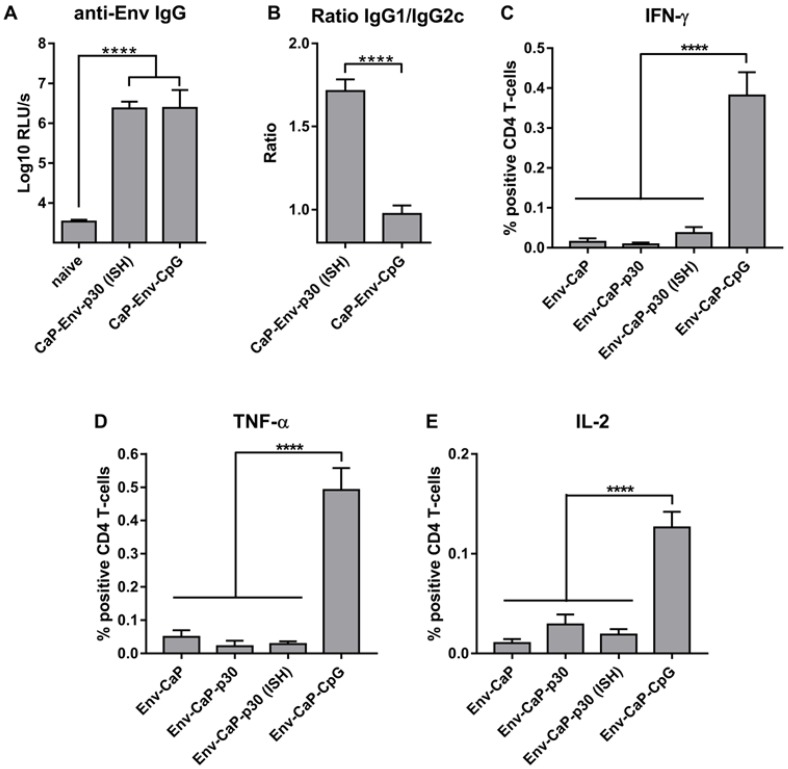
Characterization of anti-Env immune responses after heterologous ISH and CpG adjuvantation. (**A**) Env-specific antibody responses were measured in sera from wt mice primed twice with Tetanol and boosted 3 times with either Env-CaP-p30 or Env-CaP-CpG. The columns represent the mean values of six animals ± SEM. **** *p* < 0.0001; one-way ANOVA with Tukey multiple comparison post-hoc test. (**B**) Env-specific IgG1/IgG2c ratios of individual mice. The columns represent the mean values of six animals ± SEM. **** *p* < 0.0001; unpaired Student t-test. (**C**–**E**) Percentages of CD4 T-cells producing IFN-γ (**C**), TNF-α (**D**) and IL-2 (**E**) from differently immunized wt animals after in vitro re-stimulation with Env-VLPs were measured by intracellular cytokine staining. The columns represent the mean values of six animals ± SEM. **** *p* < 0.0001; one-way ANOVA with Tukey multiple comparison post-hoc test.

To compare the improvement of antibody responses by CpG and ISH side-by-side, animals previously immunized against Tetanus received either Env-CaP-p30 or Env-Cap-CpG nanoparticles. Both Env-CaP-p30 and Env-Cap-CpG induced comparable total anti-Env IgG immune responses (Figure 5A), although the anti-Env IgG subtype distribution varied between the groups (Figure 5B).

The efficacy of an HIV-1 vaccine, however, may not only depend on the strength of a protective antibody response induced, but also on the magnitude of vaccine-induced immune mechanisms increasing the susceptibility to infection. In particular, HIV-specific CD4 T-cell responses may increase the susceptibility to infection by expanding the number of activated CD4 T-cells as targets for the HIV-1 infection [1,6,7,40]. We therefore also compared CD4 T-cell responses in both vaccine groups.

In contrast to Env-CaP-p30 nanoparticles that recruit and support heterologous (non-HIV-1 specific) T-cell help (Figure 4), Env-CaP-CpG nanoparticles might induce increased Env-specific CD4 T-cell response as a result of TLR9 ligation in dendritic cells [41,42]. Indeed, the percentage of Env-specific CD4 T-cells in the spleens of immunized animals that can be reactivated in vitro after Env-VLP exposure was significantly higher in the Env-CaP-CpG immunized animals (Figure 5B–D). Reactivation of Env-specific CD4 T-cells producing Th1 pro-inflammatory [43] cytokines IFN-γ (Figure 5B) as well as TNF-α (Figure 5C) and IL-2 (Figure 5E), a potent mitogen and growth factor for CD4 T-cells, in mice from the Env-CaP-p30 (ISH) group was comparable to those in mice that received Env-CaP or Env-CaP-p30 after DPBS injections. These results clearly demonstrated that ISH does not increase expansion of Env-specific CD4 T-cells.

In non-human primate models of HIV/SIV infection, vaccinated animals with high numbers of vaccine-induced IFN-γ secreting T-cells were more susceptible to acquisition of challenge virus infection than poor T-cell responders [7,40], indicating that CpG functionalization of Env-CaP vaccines may potentially enhance the susceptibility to acquisition of HIV-1 infection in vaccinees.

Thus, the incorporation of T-helper epitopes of heterologous (non-HIV) proteins in Env-CaP nanoparticles was able to increase the magnitude of anti-Env specific IgG antibody responses by ISH without significant induction of Env-specific IFN-γ producing CD4 T-helper cells. This suggests that the ISH strategy for nanoparticle-based HIV-1 vaccines may allow to avoid induction of HIV-1 specific CD4 T-cell responses suspected to enhance the susceptibility to HIV-1 infection.

## 4. Conclusions

We demonstrated that the incorporation of T-helper cell epitopes of non-HIV proteins into CaP nanoparticles functionalized with HIV-1 Env trimers allows the Env-specific B-cells to receive T-cell help from non-HIV specific CD4 T-cells via the ISH mechanism. In a mouse model, the Env-CaP-p30 nanoparticle-based vaccine was able to improve HIV-1 Env-specific antibody responses without additional induction of HIV-specific CD4 T-cells suspected to increase the susceptibility to infection.

## Supplementary Materials

The following are available online at https://www.mdpi.com/2079-4991/9/10/1389/s1, Table S1: Characterization data of the synthesized calcium phosphate nanoparticles.
