# Supplementary material for: Calcium Phosphate Nanoparticle-Based Vaccines as a Platform for Improvement of HIV-1 Env Antibody Responses by Intrastructural Help
Source: Nanomaterials (Basel). 2019 Sep 27;9(10):1389. doi: 10.3390/nano9101389 (PMC6835376; doi:10.3390/nano9101389)
Supplement: Supplementary file 1 [file nanomaterials-09-01389-s001.pdf]

**Table S1.** Characterization data of the synthesized calcium phosphate nanoparticles[1].

| Sample | **CaP (CaP/PEI/$SiO_2$-SH)** | **Env-CaP (CaP/PEI/$SiO_2$-Env)** | **Env-CaP-p30 (CaP/PEI/p30/$SiO_2$-Env)** | **Env-CaP-CpG (CaP/PEI/CpG/$SiO_2$–Env)** |
|---|---|---|---|---|
| Solid core diameter by SEM /nm | 38 | 45 | 45 | 57 |
| *V*(one nanoparticle; only CaP)/$m^3$ | $2.85\times10^{-23}$ | $4.83\times10^{-23}$ | $4.89\times10^{-23}$ | $9.67\times10^{-23}$ |
| *m*(one nanoparticle; only CaP)/kg | $8.95\times10^{-20}$ | $1.52\times10^{-19}$ | $1.54\times10^{-19}$ | $3.04\times10^{-19}$ |
| *w*($Ca^{2+}$) by AAS/kg $m^{-3}$ | 0.068 | 0.026 | 0.032 | 0.023 |
| *w*($Ca_5(PO_4)_3OH$)/kg $m^{-3}$ | 0.171 | 0.064 | 0.079 | 0.057 |
| *N*(nanoparticles)/$m^{-3}$ | $1.91\times10^{18}$ | $4.23\times10^{17}$ | $5.14\times10^{17}$ | $1.87\times10^{17}$ |
| *w*(Env-Trimer)/kg $m^{-3}$ (85% UV-Vis Factor) | – | 0.085 | 0.085 | 0.085 |
| *w*(Env-Trimer)/kg $m^{-3}$ (Nanodrop) | – | 0.100 | 0.072 | 0.086 |
| *N*(Env-Trimer)/$m^{-3}$ | – | $3.66\times10^{20}$ | $3.66\times10^{20}$ | $3.66\times10^{20}$ |
| *m*(Env-Trimer) per nanoparticle/kg | – | $2.01\times10^{-19}$ | $1.65\times10^{-19}$ | $4.54\times10^{-19}$ |
| *N*(Env-Trimer) molecules per nanoparticle | – | 865 | 710 | 1950 |
| *N*(Env-Trimer) molecules per nanoparticle (Nanodrop) | | 1020 | 600 | 1980 |
| weight ratio of Env-Trimer loading to calcium phosphate | – | 1:1.3 | 1:1.1 | 1:1.5 |
| *w*(adjuvant)/kg $m^{-3}$ | – | – | p30:0.068 | CpG: 0.040 |
| *N*(adjuvant)/$m^{-3}$ | – | – | p30:$1.65\times10^{22}$ | CpG:$3.79\times10^{21}$ |
| *m*(adjuvant) per nanoparticle/kg | – | – | p30:$1.32\times10^{-19}$ | CpG:$2.14\times10^{-19}$ |
| *N*(adjuvant) molecules per nanoparticle | – | – | p30:$3.2\times10^{4}$ | CpG:$2.0\times10^{4}$ |
| Hydrodynamic diameter by DLS/nm (*z*-average) | 312 | 411 | 386 | 362 |
| Hydrodynamic diameter by DLS/nm (by number) | 123 | 33 | 35 | 73 |
| PDI by DLS | 0.31 | 0.43 | 0.41 | 0.38 |
| Zeta potential by DLS/mV | +27 | +20 | +23 | +27 |
| Endotoxins/EU $mL^{-1}$ | <0.1 | <0.1 | <0.1 | <0.1 |

[1]The given concentrations refer to the nanoparticle stock solutions which were then diluted and applied in the biological experiments. The estimation of the particle number concentration was based on a number of assumptions (see Materials and Methods). PDI: Polydispersity index from DLS.
